# 31. OPTIMISE-ING THE TREATMENT OF FIRST-EPISODE SCHIZOPHRENIA

**DOI:** 10.1093/schbul/sby014.126

**Published:** 2018-04-01

**Authors:** Celso Arango

**Affiliations:** Hospital General Universitario Gregorio Marañon

## Abstract

**Overall Abstract:** Even if there have been effective antipsychotic treatments for more than fifty years, the implementation of these treatments in clinical practice is still far from optimal, and a significant amount of patients with schizophrenia show poor outcomes. It is unquestionable that antipsychotics remain the first treatment option for patients with first-episode schizophrenia (FES). However, several questions still demand answer in the field. It is unclear how long we should wait before changing an antipsychotic that has not been fully effective, how long we have to wait before starting clozapine in FES, whether we can identify who will respond better to current treatments using biological markers including neuroimaging, and how to improve adherence and functional prognosis after clinical remission is achieved.

OPTiMiSE (‘Optimisation of Treatment and Management of Schizophrenia in Europe’) is an European Consortium funded under the VII Framework Program that addressed these questions in a sample of nearly 500 patients with FES or schizophreniform psychosis and minimal prior exposure to antipsychotics recruited across Europe and Israel. Within an integrative framework, the Consortium aimed at combining clinical, neuroimaging, genetic and biochemical data in a large representative sample to provide answers to these clinically relevant questions and prepare the field for personalised medication strategies as drugs with novel mechanisms of action become available. Results from this study have not yet been published and most of them will be presented for the first time in a Congress.

Based on previous results from the EUFEST study, all patients received amisulpride as a first step. For those not achieving remission after 4 weeks, OPTiMiSE compared in a randomised double-blind fashion the option of staying on amisulpride or moving to a drug with a different receptor-binding profile, olanzapine. In those not achieving remission after 6 weeks, clozapine was started. OPTiMiSE thus constitutes the first systematic, large-scale testing of the potential benefits of early switching to an antipsychotic with different characteristics in patients not achieving remission, and the application of clozapine in non-remitting patients within the first 10 weeks of treatment. OPTiMiSE also provides information on the added value of psychosocial interventions to improve treatment adherence, reduce relapses and improve functional outcomes after remission is achieved, through a 1-year follow-up randomised controlled trial testing a web-based program comprising a motivational intervention package, psychoeducation, and electronic medication alerts and updates.

OPTiMiSE also collected blood samples for patients participating in the clinical trial and a systematic sample of more than 200 standardised, high-quality, magnetic resonance imaging (MRI) images. Based on this information, OPTiMiSE examined the clinical utility and cost-effectiveness of MRI for screening of underlying organic conditions in FES. In addition, OPTiMiSE also offers novel information on how MRI alterations, genetic and biochemical markers at first presentation of schizophrenia can predict the response to subsequent treatment. For this, OPTiMiSE used two broad strategies—a combination of technology-driven (pharmacogenetics, proteomics, and metabolomics) and hypothesis-driven (neuroimaging, neurochemical, and immune-related) markers.

This symposium offers an overview of the main results obtained in the project in all these areas, with special focus on their clinical and research implications.

